# The heterogeneity among people re‐engaging in antiretroviral therapy highlights the need for a differentiated approach: results from a cross‐sectional study in Johannesburg, South Africa

**DOI:** 10.1002/jia2.26395

**Published:** 2024-12-08

**Authors:** Chipo Mutyambizi, Kate Rees, Anna Grimsrud, Rendani Ndou, Lynne S. Wilkinson

**Affiliations:** ^1^ Anova Health Institute Johannesburg South Africa; ^2^ Department of Community Health School of Public Health, University of the Witwatersrand Johannesburg South Africa; ^3^ International AIDS Society Cape Town South Africa; ^4^ Centre for Infectious Epidemiology and Research, Faculty of Health Sciences, University of Cape Town Cape Town South Africa

**Keywords:** antiretroviral therapy, health systems, HIV care, missed appointment, re‐engagement, return to care

## Abstract

**Introduction:**

Disengagement and re‐engagement with antiretroviral therapy (ART) are common in South Africa, but routine monitoring is insufficient to inform policy development. To address this gap, Anova implemented the 2020 National Adherence Guidelines’ re‐engagement standard operating procedure (re‐engagement SOP) and collected additional data to describe the characteristics of re‐engagement visits to inform HIV programmes.

**Methods:**

Between July and December 2022, we conducted a study at nine primary healthcare facilities in Johannesburg. Staff were trained on the re‐engagement SOP and provided with job aides to support implementation. Administration clerks categorized visits based on the time elapsed since the missed appointment: ≤14days and >14 days, with the latter identified as re‐engaging. For these clients, clinicians filled out “re‐engagement clinical assessment forms” that included visit dates, both clinician‐assessed and self‐reported treatment interruptions, and clinical details. Data on missed appointments and previous viral loads were extracted from medical records. The information was entered into REDCap. We present data from three out of the nine facilities, selected for their comprehensive data collection and high coverage of all re‐engaging clients.

**Results:**

A total of 2342 clients returned following a missed scheduled appointment. The majority, 1523 (65%), missed their appointments by ≤ 14 days, while 819 (35%) were >14 days late (re‐engaging). Among those re‐engaging, 635 (78%) re‐engagement clinical assessment forms were completed. A missed appointment date was available for 623 with 25% (*n* = 161) returning 2–4 weeks late, 47% (*n* = 298) 4–12 weeks and 26% (*n* = 164) >12 weeks late. Self‐reported ART interruption, available for 89% (567/635), indicated the majority (54%, *n* = 304) experienced no interruption. Clinical concerns were identified in 65 (10%) cases. A majority (79%, 504/635) had prior viral load results, with 73% (370/504) below 50 copies/ml.

**Conclusions:**

Clients frequently return to care shortly after missed appointments. Despite missing scheduled ART refill dates, many report not interrupting treatment, either having treatment on hand or sourcing ART elsewhere. Most re‐engaging clients were adherent prior to disengagement, and clinical concerns are rare. A differentiated service delivery approach, prioritizing flexibility and reduced healthcare burden, is required to support client's needs and preferences at re‐engagement.

## 1 INTRODUCTION

South Africa has the largest HIV epidemic in the world with almost 8 million people living with HIV (PLHIV) [1]. In 2023, 94% of PLHIV knew their status, 77% of those diagnosed were on antiretroviral therapy (ART) and 92% of those on ART were suppressed [2]. While the South African ART programme has rapidly expanded, significant improvements in retention are required to meet the second 95‐95‐95 target [3].

With increasing treatment interruptions, the HIV cascade is often described as cyclical, highlighting the dynamic nature of engagement with HIV care [4, 5]. While adherence to HIV treatment is critical for reducing morbidity, mortality and transmission, many PLHIV disengage from care at various stages of the continuum [4, 6]. An increasing qualitative evidence base has studied people's reasons for disengagement, re‐engagement and service preferences upon re‐engagement [7, 8, 9, 10, 11, 12]. The reasons for disengagement fall into three broad categories. The first and most common relates to healthcare attendance burden, inflexibility of ART care schedules (challenging because of mobility, competing demands like social care, time and cost) and experience (negative attitudes from staff) [7, 8, 9, 10, 12, 13]. The second relates to intra‐ and inter‐personal drivers (psychosocial reasons frequently associated with internal and external stigma) [7, 10, 11, 12]. The third encompasses clinical reasons, predominantly where the person is unwell [8, 9]. Importantly, the largest driver of disengagement appears to be mobility or migration (temporary travel or relocation) reported by 30–50% of study participants [9, 13]. Travel is often described as unplanned and beyond the control of the person required to travel either for work or social care responsibilities [7, 8, 9, 10, 11, 12, 13]. Re‐engagement is predominantly motivated by concerns over possible health deterioration and commitment to ART [8, 9, 14]. Social network support, both emotional and financial, encouraged returning to care. [8, 9].

The definition of re‐engagement varies widely within the literature, but can be defined as a client's return to care after a period of disengagement (being out of care, lost to follow‐up or interrupting treatment) [4, 15]. Some studies define re‐engagement based on the time since a missed scheduled appointment, while others define it according to the time since the last visit or contact with the health system [16].

People who disengage once are more likely to disengage again [17], and a high proportion of people presenting for ART have previous ART experience [16], highlighting the importance of improving client experience and satisfaction on return to support improved outcomes in the short to medium term. Studies have identified the importance of supportive, less judgemental, healthcare worker approaches that normalize disengagement and welcome people back into care [7, 8, 9, 17, 18]. Strong preferences have been shown for increased flexibility of ART visit schedules and associated medication refills to reduce inconvenience or clashes with family or work commitments [8, 9, 19]. These preferences are largely unmet [8, 14]. Attempts to re‐engage frequently require more than one visit or long waiting times [8, 9, 20, 21], exacerbated by unsupportive healthcare worker responses [22]. Once ART has been re‐initiated, the healthcare burden commonly intensifies with frequent facility attendance required until clinical stability is reassessed. Re‐engagement management assumes adherence challenges, and repeated enhanced adherence counselling sessions are commonly provided [23, 24].

There is limited global guidance on differentiated service delivery (DSD) models for clients re‐engaging in care. South Africa was one of the first countries to provide guiding principles and a differentiated approach. A re‐engagement algorithm was introduced in its 2020 National Adherence Guidelines Standard Operating Procedures (re‐engagement SOP) [23]. It differentiated how services are delivered to clients returning after a missed scheduled appointment based on clinical factors and duration of treatment interruption. It guided clinicians on which clients needed (i) a re‐engagement clinical assessment and increased clinical management; (ii) enhanced adherence counselling; (iii) repeat viral load measurement and the timing thereof; and (iv) reduced clinical review frequency, longer ART refills and more convenient refill collection locations to reduce health facility visit schedule burden [23]. At the time of this study, there was limited implementation of the re‐engagement SOP.

In collaboration with the Johannesburg Health District, we conducted a project to implement the re‐engagement SOP in selected facilities in Johannesburg. During the quality improvement project, we collected data to better understand the number of people returning after a missed scheduled appointment, their reasons for missing their appointment and the characteristics of re‐engagement visits to inform continued differentiated re‐engagement policy development and adaptation.

## 2 METHODS

### 2.1 Study period and setting

**Figure 1:**
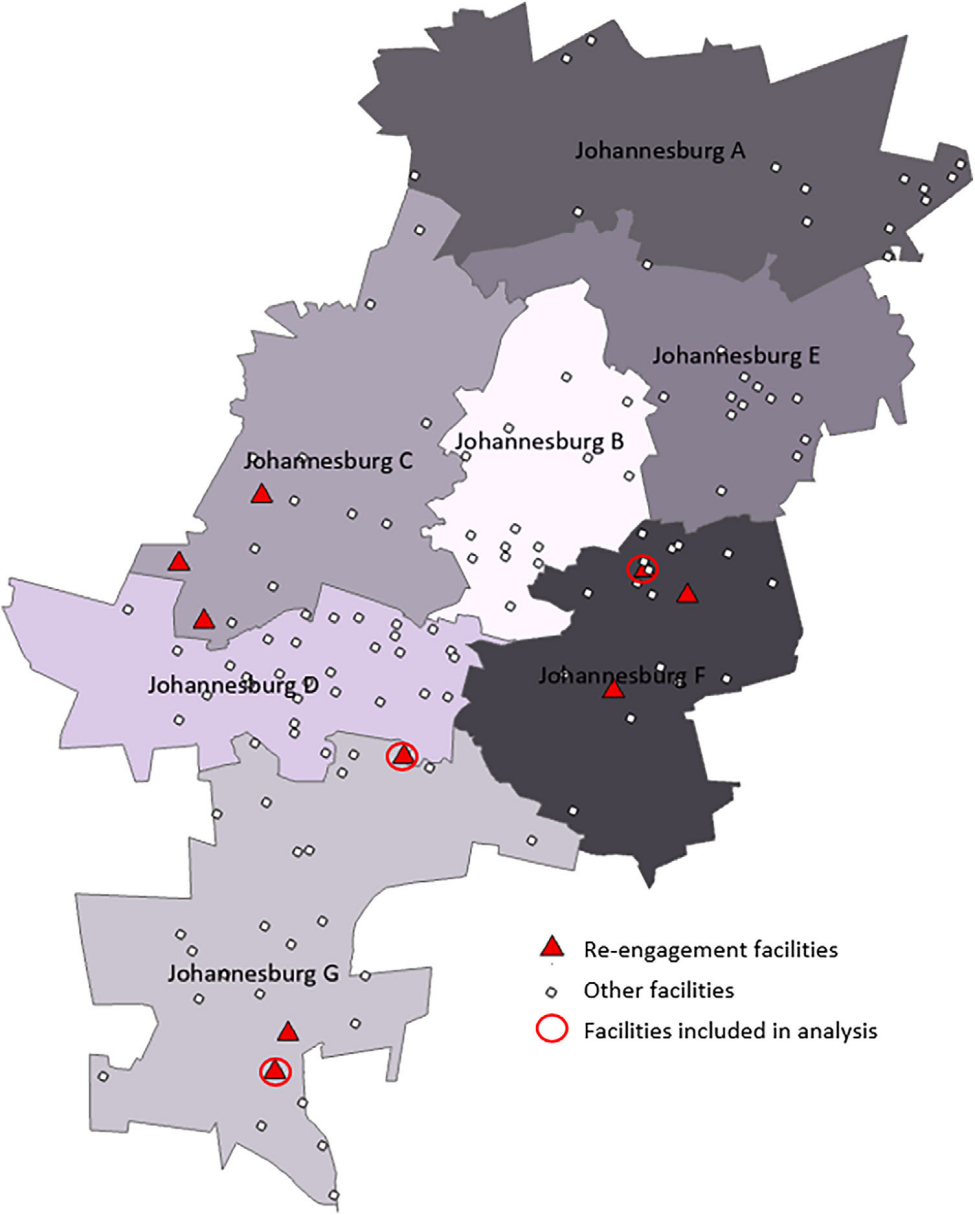
Facilities at which the project was implemented.

This study was conducted between July and December 2022 in Johannesburg, in Gauteng Province, South Africa. According to 2022 Naomi Model estimates [25], the district has a population of 6.02 million, with approximately 709,800 PLHIV, 492,000 of whom are on ART. We selected nine health facilities for this project based on the available resources for support and monitoring. Three sub‐districts within Johannesburg were selected that had no other implementation research activities. Facility selection was done in consultation with the Department of Health (DoH), including only facilities that expressed interest in the project, ensuring a balance of large and small facilities (see Figure 1).

### 2.2 Project description

Anova Health Institute is a PEPFAR/United States Agency for International Development partner to the DoH in Johannesburg. In partnership with Johannesburg District DoH, a quality improvement project was conducted to implement the re‐engagement SOP and enhance understanding of re‐engagement.

To support implementation, monitoring and evaluation, four main activities took place. (1) A training package on the re‐engagement SOP was developed, including separate job aides for clinicians, counsellors and reception staff. (2) Two stationary items were created to support implementation and facilitate data collection, endorsed by the Johannesburg DoH for use within the quality improvement project. The first was a tick sheet for administration clerks to categorize and navigate clients returning after a missed appointment. The second was a re‐engagement clinical assessment form (re‐engagement form) to be completed by clinicians during consultation. (3) Facility staff, including clinicians, counsellors or retention officers, and administration clerks, were trained on job aides, tick registers and re‐engagement forms. (4) Site‐level support was provided by a project‐trained nurse, who conducted ongoing mentoring, checked forms and provided feedback.

### 2.3 Data collection

At the reception area, individuals returning after a missed appointment were identified using appointment cards, self‐reporting or by checking clinic folders. Administration clerks completed a tick sheet indicating whether a person returned after their scheduled appointment, and whether by ≤14 days or >14 days. For this study, individuals were considered re‐engaging if they were >14 days late. All re‐engaging clients were seen by a clinician who conducted a clinical assessment and completed a re‐engagement form. Data collected included the date of re‐engagement visit, date of missed appointment, reasons for the missed appointment (optional for the client to share), clinician‐assessed and self‐reported interruption, and details identifying clinical concerns. Clinician‐assessed interruption was determined considering information from the client, time since the last visit, clinical presentation and any previous history of treatment interruptions recorded in folder. We used the dates of re‐engagement visit and the missed appointment to calculate time to return. For clients with a missing scheduled visit date, a search was conducted in TIER.Net (the national public sector facility HIV database) [26] to capture the missing dates. Pre‐disengagement viral load details were extracted from clinic folders. We calculated months since the last viral load using the date of re‐engagement visit and the date of the last viral load result. A copy of the completed re‐engagement form was placed in the client folder and a second copy was captured into REDCap [27].

### 2.4 Data analysis

Data were imported into STATA version 18 and analysed descriptively. Poor tick register and re‐engagement form completion occurred at six of the nine health facilities, risking distortion of primary outcomes due to a non‐representative subgroup of re‐engaging clients at these facilities. Therefore, we restricted the final sample for analysis to three facilities (Figure 1), where the number of clients recorded on re‐engagement forms as returning 4 weeks after a scheduled appointment was at least 95% of the number reported as re‐engaging from TIER.Net, according to PEPFAR requirements. PEPFAR defines return to treatment as “ART clients with no clinical contact (or ARV drug pick‐up) for greater than 28 days since their last expected contact who restarted ARVs within the reporting period” (MER data indicator—TX_RTT) [28]. District Health Information System data reflects approximately 62,000 people on ART by 31 January 2024 in all nine facilities and 39,000 in the three facilities included in the final sample.

### 2.5 Ethical clearance

Ethical approval was obtained from the Human Sciences Research Council (HSRC) REC 3/22/08/18 and the Johannesburg District Research Committees.

## 3 RESULTS

**Figure 2:**
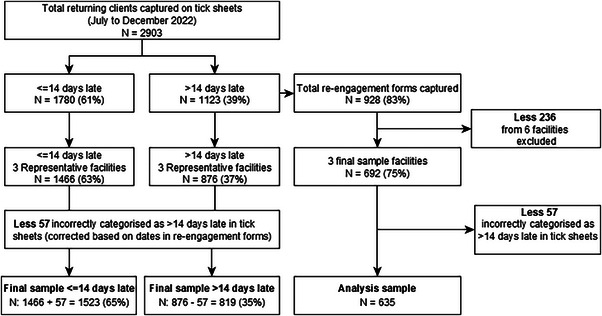
Flow chart for final analytic sample.

Administration clerks recorded 2903 individuals returning after a scheduled ART appointment across all nine facilities during the 6‐month study period (Figure 2). Of these, 61% (1780/2903) missed their appointment by 14 days or less, while 39% (1123/2903) exceeded the 14‐day threshold and were classified as re‐engaging. When limiting the analysis to the three representative facilities, 63% (1466/2342) were listed as late by ≤14 days, and 37% (876/2342) by >14 days.

### 3.1 Final sample

A total of 692 re‐engagement forms were captured. The timing of return to facility indicated that the administration clerk completed the tick sheet had incorrectly categorized 57 clients as re‐engaging when they were <14 days late. In total, 65% (1523/2342) of clients returned 14 days or less late, and 35% (819/2342) returned more than 14 days after their scheduled missed appointment. Our final analytic sample of re‐engaging clients with completed re‐engagement forms was 635 clients.

### 3.2 Time taken to return after a missed scheduled appointment

Among those re‐engaging with a completed re‐engagement form (*n* = 635), 25% (*n* = 161) were 2–4 weeks late, 47% (*n* = 298) were 4–12 weeks late and 26% (*n* = 164) were more than 12 weeks late. For 12 individuals (2%), their last scheduled appointment was not recorded on the form or in TIER.Net, leaving the exact number of weeks since their last visit unknown. The proportion of return visits classified as re‐engaging and the time to return varied by facility, as shown in Table 1 (Table S1 for all nine facilities). Facility 1 was an outlier, with fewer individuals returning within 14 days and a higher proportion of re‐engaging clients taking longer to return.

**Table 1:** Time to return after a missed scheduled visit by facility

Variables	Facility 1	Facility 2	Facility 3	Total
	*N* (%)	*N* (%)	*N* (%)	*N* (%)
**Time since scheduled visit Tick sheets**	(*N* = 64)	(*N* = 592)	(*N* = 1686)	(*N* = 2342)
≤14 days	10 (16)^a^	446 (75)	1067 (63)	1523 (65)
>14 days (re‐engaging)	54 (84)^a^	146 (25)	619 (37)	819 (35)
**Re‐engagement forms**				
>14 days (re‐engaging)	*N* = 72	*N* = 98	*N* = 465	*N* = 635
2–4 weeks	10 (14)	31 (32)	120 (26)	161 (25)
4–12 weeks	29 (40)	51 (52)	218 (47)	298 (47)
12+ weeks	31 (43)	13 (13)	120 (26)	164 (26)
*Missing*	2 (3)	3 (3)	7 (2)	12 (2)

^a^ Facility 1 under reported on tick sheets. See the risk of under reporting addressed in limitations in Discussion section below.

### 3.3 Reasons for missing a scheduled appointment

Among those who provided a reason for returning after their scheduled appointment (64%, 405/635), the most common reasons were being out of town (42%, 172/413) and being at work (24%, 99/413), as shown in Table 2 (see Table S2 for all nine facilities). Almost all individuals had their last visit at the same facility where their missed appointment was scheduled (99%, 602/610).

**Table 2:** Reasons for missed visit

Variables	Facility 1	Facility 2	Facility 3	Total
Number of reasons provided	15 (4)	78 (19)	320 (77)	413
Proportion of re‐engaging clients with a reason provided	21% (15/72)	80% (78/98)	67% (312/465)	64% (405/635)
**Reason for missed visit**				
Out of town	7 (47)	17 (22)	148 (46)	172 (42)
I was at work	5 (33)	24 (31)	70 (22)	99 (24)
I misplaced my card/didn't know	0 (0)	14 (18)	18 (6)	32 (8)
Family obligation	1 (7)	12 (15)	15 (5)	28 (7)
I was feeling sick or personal health issues	0 (0)	4 (5)	21 (7)	25 (6)
I still had medication	1 (7)	1 (1)	18 (6)	20 (5)
I forgot	0 (0)	3 (4)	9 (3)	12 (3)
I was in prison	1 (7)	1 (1)	6 (2)	8 (2)
Other	0 (0)	2 (3)	15 (5)	17 (4)
*Total*	15 (100)	78 (100)	320 (100)	413 (100)

### 3.4 Description of treatment interruptions

Among those with the field completed, 54% (304/567) self‐reported no treatment interruption, as shown in Table 3. Despite being late, individuals reported utilizing stock on hand. Independent clinician assessment of treatment interruption was similar, with 53% (221/419) of those with a documented clinical assessment considered not to have interrupted treatment. Those late by more than 12 weeks were more likely to self‐report a treatment interruption (67%, 97/144) compared to those late by 2−4 weeks (38%, 57/151).

**Table 3:** Description of people who re‐engaged after a missed scheduled appointment at study clinics by weeks since scheduled appointment

Variables	Time since scheduled visit			Total
	2−4 weeks	4−12 weeks	12+ weeks	
	*N* = 161 (25)	*N* = 298 (47)	*N* = 164 (26)	*N* = 635
	*N* (%)	*N* (%)	*N* (%)	*N* (%)
**Patient presentation**				
Well	155 (96)	284 (95)	149 (91)	599 (94)
Unwell^a^	5 (3)	13 (4)	15 (9)	34 (5)
*Missing*	1 (1)	1 (0)	0 (0)	2 (0)
*Total*	161 (100)	298 (100)	164 (100)	635 (100)
**Self‐report treatment interruption**				
Yes	57 (35)	102 (34)	97 (59)	263 (41)
No	94 (58)	163 (55)	47 (29)	304 (48)
*Missing*	10 (6)	33 (11)	20 (12)	68 (11)
*Total*	161 (100)	298 (100)	164 (100)	635 (100)
**Clinician assessed treatment interruption**				
Yes	38 (24)	83 (28)	74 (45)	198 (31)
No	63 (39)	115 (39)	38 (23)	221 (35)
*Missing*	60 (37)	100 (34)	52 (32)	216 (34)
*Total*	161 (100)	298 (100)	164 (100)	635 (100)
**Clinician assessed clinical concerns**				
Yes	9 (6)	35 (12)	21 (13)	65 (10)
No	114 (71)	211 (71)	113 (69)	448 (71)
*Missing*	38 (24)	52 (17)	30 (18)	122 (19)
*Total*	161 (100)	298 (100)	164 (100)	635 (100)
**Months since last viral load at re‐engagement**				
< 6 months	52 (32)	88 (30)	27 (16)	171 (27)
6−12 months	53 (33)	72 (24)	42 (26)	167 (26)
12+ months	24 (15)	57 (19)	52 (32)	136 (21)
*Missing*	32 (20)	81 (27)	43 (26)	161 (25)
*Total*	161 (100)	298 (100)	164 (100)	635 (100)
**Last viral load value**				
<50 copies/ml	101 (63)	186 (62)	77 (47)	370 (58)
50−399 copies/ml	21 (13)	31 (10)	27 (16)	79 (12)
400−999 copies/ml	4 (2)	2 (1)	5 (3)	11 (2)
>1000 copies/ml	6 (4)	24 (8)	13 (8)	44 (7)
*Missing*	29 (18)	55 (18)	42 (26)	131 (21)
*Total*	161 (100)	298 (100)	164 (100)	635 (100)

^a^ Unwell includes symptomatic and red flag sypmtoms

### 3.5 Clinical status of those returning to care

The majority of re‐engaging individuals reported having no symptoms, or feeling well (94%, 599/635), with clinical concerns identified by clinicians in 10% (65/635), as shown in Table 3 (Table S3 for all nine facilities). A higher proportion reported being unwell or symptomatic (9%) or were assessed as having clinical concerns (13%) in the group that had missed their appointment by more than 12 weeks. A pre‐disengagement viral load result was available for 79% of re‐engaging individuals (504/635). The majority (58%, 370/635) were suppressed with a viral load <50 copies/ml, 12% had a viral load of 50–399 copies/ml (79/635), 2% had a viral load of 400–999 copies/ml (11/635) and 7% had a viral load >1000 copies/ml (44/635).

## 4 DISCUSSION

We found many people returning within 2 weeks of a missed scheduled ART appointment. Among those re‐engaging, defined as returning after 2 weeks, two‐thirds returned to care within 12 weeks. Despite missing their appointment by over 2 weeks, the majority of re‐engaging clients were adherent with suppressed viral loads prior to disengagement and self‐reported not interrupting treatment despite late return. Clinical concerns were uncommon.

The definition of re‐engagement in this study was chosen to align with guiding national policy, including tracing protocols and less‐intensive DSD model exit criteria. While the definition is likely conservative, many other definitions would not have identified the majority of people in our study, despite their high numbers and impact on constrained health systems and overburdened providers [16]. To our knowledge, this is the first study to quantify the number of ART clients returning within 28 days of a missed scheduled appointment and their time to return.

Our study showed that many people missed a scheduled appointment but returned within 3 months. Previous research has demonstrated a correlation between missed appointments and treatment interruptions, and poorer long‐term retention and viral suppression [29, 30]. However, the extent to which experiences during return visits contribute to these outcomes remains unknown. Given the recurrent emphasis in qualitative studies on the importance of client experience and service delivery satisfaction on return [8, 9, 18], it is plausible that poor experiences contribute to adverse long‐term outcomes.

In our study, not all people who returned after a missed appointment had interrupted ART. Among those late by more than 2 weeks, many self‐reported not having interrupted treatment, with ART still on hand from previous dispenses or having sourced ART elsewhere. Clients are known to self‐manage when refill collection is not possible. When unplanned travel occurs, the clinic in the area of travel commonly provides a month refill to ensure a client does not run out of medication. Clients also borrow medication from their social network and replace when they collect their refill [9]. There are also opaque black‐market systems that sell emergency refills [31]. On assessment, clinicians also considered many re‐engaging clients not to have interrupted. While self‐report may not be a reliable measure due to susceptibility to social desirability and affirmation biases [32] and clinician assessment has also been shown to be flawed [33], it likely indicates some level of self‐care resourcefulness that needs to be considered. Importantly, the majority of those returning to care were adherent, as indicated by suppressed viral loads before their scheduled appointment and were not unwell on return. Unsurprisingly, self‐report of treatment interruption and being assessed as clinically unwell was more common the later the return.

The top two reported reasons for missing appointments were being out of town and being at work. Mobility/being away from home and busy work schedules have been similarly reported in other studies [9, 13]. A third of patients in a study by Bisnauth et al. reported mobility as a reason for disengagement from care [9]. Services need to accommodate people's life experiences, and not penalize them for having competing priorities. Appointment dates should be set in consultation with clients wherever possible, communicated clearly and allow for some flexibility.

Our collated findings underscore the importance of mitigating the risk that attending a health facility shortly after a scheduled appointment turns into a prolonged interruption or repeated interruptions due to a poor experience at return, or a lack of responsiveness to visit burdens or inflexibility. Service differentiation should align with service delivery preferences on return, as identified in prior qualitative research [8, 9], providing more flexibility and less burdensome visit schedules when clinically safe. Furthermore, enhanced adherence counselling should not be mandated for all patients re‐engaging, as it may not always be warranted.

Considering the high number of people who returned shortly after a missed scheduled appointment, coupled with the escalating clinical implications as the duration since missed scheduled appointment extends, clinical presentation at re‐engagement and the interval from scheduled appointment to return should guide service delivery differentiation.

South Africa introduced new clinical and service delivery guidelines in 2023, with a re‐engagement algorithm which was partly informed by this data [24]. Guiding principles prioritize a welcoming, non‐judgemental healthcare provider approach with immediate ART resupply and prohibit punitive actions. People who self‐identify as well, are not on tuberculosis (TB) treatment, and return within 28 days of their scheduled appointment date, continue their management in South Africa's less‐intensive DSD models (longer ART refills with collection from external pick‐up points, fast track facility pick‐up points or adherence clubs) or should be assessed for eligibility and enrolled. All other returnees require clinical assessment. For those assessed to be well and less than 90 days late, immediate assessment for (re)enrolment in South Africa's less‐intensive DSD models is recommended. Alternatively, their visit schedule does not intensify but resets to 3‐monthly with an aligned multi‐month ART refill. For those more than 90 days late, a CD4 is taken to identify and recall clients with advanced HIV disease (AHD). Returnees receive 3 months of ART to align with their follow‐up viral load. If suppressed, they are assessed for less‐intensive DSD. Those unwell, on TB treatment, identified with AHD or with a pre‐disengagement elevated viral load, receive more focused clinical management. This allows for increased visit spacing and aligned longer ART refills when there is no clinical reason for more frequent attendance. An enhanced adherence counselling session is recommended for people struggling with intra‐ and inter‐personal challenges with adherence. This DSD approach to re‐engagement aims to improve return experience and rapidly reduce visit burdens for both client and healthcare providers.

The strengths of our study are: firstly, we collected primary data on the number of people returning after a missed appointment and time to return. Secondly, we collected several variables not collected in routine monitoring systems for all people returning 14 days or more after their missed scheduled visit. Thirdly, using a structured re‐engagement form guided clinicians to cover key important aspects of the consultation in a standardized way. Lastly, except for treatment interruption and reasons for missing the scheduled appointment, the study did not rely solely on self‐reported data.

This study, embedded in public sector implementation, offers a more accurate reflection of real‐world outcomes but also has limitations. Our main limitation was inconsistent fidelity to implementation and data collection. To minimize bias, we reported outcomes only from three facilities that achieved a data collection rate of at least 95% of individuals independently reported to PEPFAR as re‐engaging, using data from South Africa's public sector HIV database—TIER.net. Since PEPFAR defines re‐engagement starting at 28 days post‐missed appointment, we could not ascertain whether individuals returning within 28 days were underreported. In particular, Facility 1 reported fewer individuals within this timeframe, which suggests possible underreporting at this site. However, any unrecorded returns within 28 days would likely increase the proportion of early returns observed.

Limiting the final analysis to the three facilities with high data collection fidelity may have introduced selection bias, as these sites may not be fully representative of all nine facilities included in the study. This could potentially skew the findings, particularly if these facilities had distinct characteristics or client behaviours. Nevertheless, we deemed this approach less prone to bias than including data from facilities with incomplete reporting for all re‐engaging clients, which would have further compromised the reliability of results. To provide a broader perspective, we included result tables from all nine facilities in a Supplementary Appendix for comparative analysis.

Our study did not collect data on age, gender or sex, so we could not present findings disaggregated by these demographics. Relying on clinical records as the primary data source led to missing data, a common challenge in such facilities [34]. The pattern of missing data showed no consistency across the facilities analysed. Another limitation is our focus on individuals who returned to care, excluding those who missed their appointment and did not return within the study timeframe.

Our findings, derived from South African public sector facilities with both small and large ART cohorts adhering to national guidelines, may not be fully generalizable, given the small number of facilities included, their exclusive urban setting within a single province and their purposive selection. Replicating this study in other settings would be of interest.

Future research is required to evaluate the impact of providing a DSD approach on re‐engagement with more flexibility and a less onerous visit schedule burden while meeting specific clinical needs. This should include client experience and satisfaction, longer‐term retention and viral suppression outcomes. South Africa's newly introduced national re‐engagement algorithm offers a unique opportunity for such evaluation.

## 5 CONCLUSIONS

While many people in our study did not attend their scheduled ART appointment date, the majority returned within 2 weeks, and over three‐quarters returned within 3 months. Despite missing a scheduled ART refill date, many, especially those returning within a month, reported not interrupting ART, with viral load history confirming adherence prior to their missed appointment. Clinical concerns arising from missed appointments were rare, as indicated by clinical assessments upon re‐engagement. Tailoring service delivery to be more client‐centred and responsive to the needs and preferences of people who miss appointments can reduce their healthcare burden, enhance their return experience and support long‐term retention and viral suppression.

## COMPETING INTERESTS

The authors declare that they have no competing interests.

## AUTHORS’ CONTRIBUTIONS

*Conceptualization*: CM, LSW and KR. *Data collection*: CM and RN. *Data analysis*: CM, critical reviews of data analysis LSW and KR. *Writing—original draft*: CM. *Subsequent drafts*: CM and LSW. *Writing—reviewing and editing*: LSW, AG and KR. *Project administration*: CM. All authors provided approval for the version to be published.

## FUNDING

This work is supported by IAS—the International AIDS Society with financial support from the Bill & Melinda Gates Foundation (INV‐00261 and INV‐047567). This study is also made possible by the support of the US President's Emergency Plan for AIDS Relief (PEPFAR) through the United States Agency for International Development (USAID) under Cooperative Agreement number 674‐A‐12‐00015 to the Anova Health Institute.

## DISCLAIMER

The contents are the responsibility of Anova Health Institute and do not necessarily reflect the views of USAID or the United States Government.

## Supporting information




**Table S1**: Time to return after a missed scheduled visit by facility
**Table S2**: Reasons for missed visit
**Table S3**: Description of people who re‐engaged after a missed scheduled appointment at study clinics by weeks since scheduled appointment

## Data Availability

The data that support the findings of this study are available from the corresponding author upon reasonable request.
