# Supplementary material for: The heterogeneity among people re‐engaging in antiretroviral therapy highlights the need for a differentiated approach: results from a cross‐sectional study in Johannesburg, South Africa
Source: J Int AIDS Soc. 2024 Dec 8;27(12):e26395. doi: 10.1002/jia2.26395 (PMC11625505; doi:10.1002/jia2.26395)
Supplement: Supplementary file 1 — Table S1: Time to return after a missed scheduled visit by facility Table S2: Reasons for missed visit Table S3: Description of people who re‐engaged after a missed scheduled appointment at study clinics by weeks since scheduled appointment [file JIA2-27-e26395-s001.docx]

Supplementary tables

Table 1: Time to return after a missed scheduled visit by facility.

| **Variables** | **Fac1** | **Fac2** | **Fac3** | **Fac4** | **Fac5** | **Fac6** | **Fac7** | **Fac8** | **Fac9** | **All 9 facilities** | **Facility 1, 2 and 3** |
| --- | --- | --- | --- | --- | --- | --- | --- | --- | --- | --- | --- |
|  | **N (%)** | **N (%)** | **N (%)** | **N (%)** | **N (%)** | **N (%)** | **N (%)** | **N (%)** | **N (%)** | **N (%)** | **N (%)** |
| Number on ART Jan 2024 (DHIS) | 1947 | 5055 | 32052 | 2957 | 2603 | 786 | 10441 | 1323 | 4644 | 61808 | 39054 |
| **Time since scheduled visit** |  |  |  |  |  |  |  |  |  |  |  |
| **Tick sheets** | N=64 | N=592 | N=1686 | N=96 | N=30 | N=224 | N=0 | N=63 | N=148 | N=2903* | N=2342 |
| ≤14 days | 10 (16) | 446 (75) | 1067 (63) | 20 (21) | 8 (27) | 181 (81) | 0 (0) | 45 (71) | 68 (46) | 1845 (64) | 1523 (65) |
| >14 days (re-engaging) | 54 (84) | 146 (25) | 619 (37) | 76 (79) | 22 (73) | 43 (19) | 0 (0) | 18 (29) | 80 (54) | 1058 (36) | 819 (35) |
| **Re-engagement forms** |  |  |  |  |  |  |  |  |  |  |  |
| >14 days (re-engaging) | N=72 | N=98 | N=465 | N=15 | N=35 | N=36 | N=55 | N=25 | N=62 | N=863 | N=635 |
| 2-4 weeks | 10 (14) | 31 (32) | 120 (26) | 1 (7) | 1 (3) | 8 (22) | 8 (15) | 5 (20) | 5 (8) | 189 (22) | 161 (25) |
| 4-12 weeks | 29 (40) | 51 (52) | 218 (47) | 1 (7) | 9 (26) | 10 (28) | 14 (25) | 14 (56) | 19 (31) | 365 (42) | 298 (47) |
| 12+ weeks | 31 (43) | 13 (13) | 120 (26) | 11 (73) | 9 (26) | 5 (14) | 16 (29) | 4 (16) | 14 (23) | 223 (26) | 164 (26) |
| *Missing* | 2 (3) | 3 (3) | 7 (2) | 2 (13) | 16 (46) | 13 (36) | 17 (31) | 2 (8) | 24 (39) | 86 (10) | 1. (2) |

*The table reflects the final sample. Administration clerks tick sheet had incorrectly categorized 65 clients as re-engaging clients when these clients were <14 days late.

**Table 2: Reasons for missed visit**

| **Variables** | **Fac1** | **Fac2** | **Fac3** | **Fac4** | **Fac5** | **Fac6** | **Fac7** | **Fac8** | **Fac9** | **All 9 facilities** | **Facility 1, 2 and 3** |
| --- | --- | --- | --- | --- | --- | --- | --- | --- | --- | --- | --- |
| Number of reasons provided | 15 (3) | 78 (15) | 320 (62) | 4 (1) | 13 (3) | 24 (5) | 21 (4) | 10 (2) | 32 (6) | 517 (100) | 413 (80) |
| Proportion of re-engaging clients with a reason provided | 21% (15/72) | 80% (78/98) | 67% (312/465) | 27% (4/15) | 37% (13/35) | 67% (24/36) | 38% (21/55) | 40% (10/25) | 52% (32/62) | 59% (508/863) | 64% (405/635) |
| **Reason for missed visit** | **N (%)** | **N (%)** | **N (%)** | **N (%)** | **N (%)** | **N (%)** | **N (%)** | **N (%)** | **N (%)** | **N (%)** | **N (%)** |
| Out of town | 7 (47) | 17 (22) | 148 (46) | 3 (75) | 3 (23) | 8 (33) | 11 (52) | 3 (30) | 12 (38) | 212 (41) | 172 (42) |
| I was at work | 5 (33) | 24 (31) | 70 (22) | 0 (0) | 2 (15) | 3 (13) | 0 (0) | 4 (40) | 6 (19) | 114 (22) | 99 (24) |
| I misplaced my card/didn’t know | 0 (0) | 14 (18) | 18 (6) | 0 (0) | 0 (0) | 2 (8) | 1 (5) | 1 (10) | 0 (0) | 36 (7) | 32 (8) |
| Family obligation | 1 (7) | 12 (15) | 15 (5) | 0 (0) | 0 (0) | 3 (13) | 2 (10) | 0 (0) | 2 (6) | 35 (7) | 28 (7) |
| I was feeling sick or personal health issues | 0 (0) | 4 (5) | 21 (7) | 0 (0) | 4 (31) | 2 (8) | 1 (5) | 0 (0) | 2 (6) | 34 (7) | 25 (6) |
| I still had medication | 1 (7) | 1 (1) | 18 (6) | 1 (25) | 1 (8) | 1 (4) | 4 (19) | 0 (0) | 1 (3) | 28 (5) | 20 (5) |
| I forgot | 0 (0) | 3 (4) | 9 (3) | 0 (0) | 2 (15) | 3 (13) | 2 (10) | 0 (0) | 6 (19) | 25 (5) | 12 (3) |
| I was in prison | 1 (7) | 1 (1) | 6 (2) | 0 (0) | 0 (0) | 1 (4) | 0 (0) | 0 (0) | 1 (3) | 10 (2) | 8 (2) |
| Other | 0 (0) | 2 (3) | 15 (5) | 0 (0) | 1 (8) | 1 (4) | 0 (0) | 2 (20) | 2 (6) | 23 (4) | 17 (4) |
| *Total* | 15(100) | 78(100) | 320(100) | 4 (100) | 13 (100) | 24 (100) | 21 (100) | 10 (100) | 32 (100) | 517 (100) | 413(100) |
| **Last scheduled visit** |  |  |  |  |  |  |  |  |  |  |  |
| This facility | 65 (90) | 95 (97) | 442 (95) | 14 (93) | 23 (66) | 30 (83) | 46 (84) | 22 (88) | 55 (89) | 792 (92) | 602 (95) |
| Other facility (transfer in) | 1 (1) | 0 (0) | 7 (2) |  | 6 (17) | 3 (8) | 7 (13) | 3 (12) |  | 27 (3) | 8 (1) |
| *Missing* | 6 (8) | 3 (3) | 16 (3) | 1 (7) | 6 (17) | 3 (8) | 2 (4) |  | 7 (11) | 44 (5) | 25 (4) |

Table 3: Description of people who re-engaged after a missed scheduled appointment at study clinics by weeks since scheduled appointment

|  | **All 9 facilities** | | | | **Facility 1, 2 and 3** | | | |
| --- | --- | --- | --- | --- | --- | --- | --- | --- |
| **Variables** | **Time since scheduled visit** | | | **Total** | **Time since scheduled visit** | | | **Total** |
|  | 2 to 4 weeks | 4 to 12 weeks | 12+ weeks |  | 2 to 4 weeks | 4 to 12 weeks | 12+ weeks |  |
|  | N = 189 (22) | N = 365 (42) | N = 223 (26) | N = 863 | N=161 (25) | N=298 (47) | N=164 (26) | N = 635 |
|  | N (%) | N (%) | N (%) |  | N (%) | N (%) | N (%) | N (%) |
| **Patient presentation** |  |  |  |  |  |  |  |  |
| Well | 183 (97) | 347 (95) | 200 (89) | 805 (93) | 155 (96) | 284 (95) | 149 (91) | 599 (94) |
| Unwell* | 5 (3) | 15 (4) | 19 (9) | 46 (5) | 5 (3) | 13 (4) | 15 (9) | 34 (5) |
| *Missing* | 1 (1) | 3 (1) | 4 (2) | 12 (1) | 1 (1) | 1 (0) | 0 (0) | 2 (0) |
| *Total* | 189 (100) | 365 (100) | 223 (100) | 863 (100) | 161 (100) | 298 (100) | 164 (100) | 635 (100) |
| **Self-report treatment interruption** |  |  |  |  |  |  |  |  |
| Yes | 72 (38) | 137 (38) | 137 (61) | 378 (44) | 57 (35) | 102 (34) | 97 (59) | 263 (41) |
| No | 104 (55) | 181 (50) | 56 (25) | 352 (41) | 94 (58) | 163 (55) | 47 (29) | 304 (48) |
| *Missing* | 13 (7) | 47 (13) | 30 (13) | 133 (15) | 10 (6) | 33 (11) | 20 (12) | 68 (11) |
| *Total* | 189 (100) | 365 (100) | 223 (100) | 863 (100) | 161 (100) | 298 (100) | 164 (100) | 635 (100) |
| **Clinician assessed treatment interruption** |  |  |  |  |  |  |  |  |
| Yes | 39 (21) | 99 (27) | 93 (42) | 245 (28) | 38 (24) | 83 (28) | 74 (45) | 198 (31) |
| No | 74 (39) | 129 (35) | 49 (22) | 265 (31) | 63 (39) | 115 (39) | 38 (23) | 221 (35) |
| *Missing* | 76 (40) | 137 (37) | 81 (36) | 353 (41) | 60 (37) | 100 (34) | 52 (32) | 216 (34) |
| *Total* | 189 (100) | 365 (100) | 223 (100) | 863 (100) | 161 (100) | 298 (100) | 164 (100) | 635 (100) |
| **Clinician assessed clinical concerns** |  |  |  |  |  |  |  |  |
| Yes | 10 (5) | 40 (11) | 31 (14) | 93 (11) | 9 (6) | 35 (12) | 21 (13) | 65 (10) |
| No | 135 (71) | 247 (68) | 145 (65) | 565 (65) | 114 (71) | 211 (71) | 113 (69) | 448 (71) |
| *Missing* | 44 ()23 | 78 (21) | 47 (21) | 205 (24) | 38 (24) | 52 (17) | 30 (18) | 122 (19) |
| *Total* | 189 (100) | 365 (100) | 223 (100) | 863 (100) | 161 (100) | 298 (100) | 164 (100) | 635 (100) |
| **Months since last VL at re-engagement** |  |  |  |  |  |  |  |  |
| < 6 months | 59 ()31 | 102 (28) | 35 (16) | 212 (25) | 52 (32) | 88 (30) | 27 (16) | 171 (27) |
| 6 to 12 months | 63 (33) | 96 (26) | 48 (22) | 223 (26) | 53 (33) | 72 (24) | 42 (26) | 167 (26) |
| 12+ months | 32 (17) | 63 (17) | 75 (34) | 181 (21) | 24 (15) | 57 (19) | 52 (32) | 136 (21) |
| *Missing* | 35 (19) | 104 (28) | 65 (29) | 247 (29) | 32 (20) | 81 (27) | 43 (26) | 161 (25) |
| *Total* | 189 (100) | 365 (100) | 223 (100) | 863 (100) | 161 (100) | 298 (100) | 164 (100) | 635 (100) |
| **Last viral load value** |  |  |  |  |  |  |  |  |
| <50 copies/ml | 121 (64) | 220 (60) | 102 (46) | 473 (55) | 101 (63) | 186 (62) | 77 (47) | 370 (58) |
| 50 to 399 copies/ml | 24 (13) | 38 (10) | 32 (14) | 96 (11) | 21 (13) | 31 (10) | 27 (16) | 79 (12) |
| 400 to 999 copies/ml | 4 (2) | 4 (1) | 6 (3) | 16 (2) | 4 (2) | 2 (1) | 5 (3) | 11 (2) |
| >1000 copies/ml | 6 (3) | 29 (8) | 17 (8) | 63 (7) | 6 (4) | 24 (8) | 13 (8) | 44 (7) |
| *Missing* | 34 (18) | 74 (20) | 66 (30) | 215 (25) | 29 (18) | 55 (18) | 42 (26) | 131 (21) |
| *Total* | 189 (100) | 365 (100) | 223 (100) | 863 (100) | 161 (100) | 298 (100) | 164 (100) | 635 (100) |
